# Examining the relationship between perceived social support and prenatal distress in pregnant women

**DOI:** 10.3389/fpsyt.2025.1514249

**Published:** 2025-09-22

**Authors:** Somayae Abdollahi Sabet, Samira Ahmadi, Zahra Pakian, Azam Maleki

**Affiliations:** ^1^ Social Determinants of Health Research Center, Health and Metabolic Diseases Research Institute, Zanjan University of Medical Sciences, Zanjan, Iran; ^2^ Department of Community Medicine, Faculty of Medicine, Zanjan University of Medical Sciences, Zanjan, Iran

**Keywords:** social support, psychological distress, perinatal care, women’s health, perinatal distress

## Abstract

**Background:**

Understanding the link between perceived social support and prenatal distress is vital for improving maternal mental health during pregnancy.

**Objective:**

To examine the relationship between perceived social support and prenatal distress among pregnant women.

**Methods:**

A cross-sectional study was conducted on 220 pregnant women at Ayatollah Mousavi Hospital in Zanjan City in 2023. Participants were selected through a convenience sampling method, and data were collected using the Prenatal Distress and social support questionnaires. The analysis involved Spearman’s rank correlation, the Mann–Whitney U test, the Kruskal–Wallis test, and backward conditional logistic regression with median cut-off points, all at a 95% confidence level.

**Results:**

The average perinatal distress score was 39.38 (SD = 6.04), and the average social support score was 45.66 (SD = 10.91). Total social support was negatively correlated with overall prenatal distress (r = -0.355), childbirth and the baby-related distress (r =-0.472), and emotional/relational issues(r = -0.119), but not with body image-related distress (r = 0.090, p = 0.35). Younger, higher-educated women with more children reported lower distress. Additionally, women aged 18-25, with more children, low income, who were housewives, or had a gestational age of 29–42 weeks, had higher social support scores (p < 0.01).

**Conclusion:**

Both perinatal distress and perceived social support were observed at moderate levels among the study population. Parity emerged as the only independent predictor for both prenatal distress and social support, with primiparous women exhibiting higher distress levels, while multiparous women reported higher perceived social support. These findings underscore the importance of developing tailored interventions that address the distinct needs of women according to their parity status.

## Introduction

Motherhood is a transformative journey beginning with pregnancy that brings profound changes in a woman’s relationships with partners, family, friends, and the broader social environment. During this transition, many women experience emotional fluctuations such as anxiety, stress, and self-doubt (1). The perinatal period, spanning from pregnancy to 12 months postpartum, is characterized by significant social, emotional, biological, and psychological changes (2). Self-reported anxiety symptoms were observed in 18.2% of women during the first trimester, rising slightly to 19.1% in the second trimester, and reaching 24.6% in the third trimester. Clinically diagnosed anxiety disorders were present in 15.2% of the population, with generalized anxiety disorder accounting for 4.1% (3). Stress and anxiety during pregnancy are increasingly recognized as critical factors influencing adverse maternal and fetal outcomes. Recent evidence indicates that elevated maternal stress contributes to complications such as prenatal depression, hypertension, and immune dysregulation, thereby adversely impacting maternal health (4, 5). Prenatal stress can lead to a wide range of adverse outcomes in offspring, including neurodevelopmental disorders, emotional dysregulation, cognitive impairments, mood disorders, and an increased risk of various psychopathological conditions. Moderating factors such as the severity of maternal stress, maternal mental health status, socioeconomic conditions, availability of social support, and exposure to early-life adversity can significantly modulate the effects of prenatal stress on offspring outcomes (6) A study by Figen Alp Yilmaz et al. demonstrated that prenatal distress levels among pregnant women are influenced by various factors, including geographic region, lack of spousal support, and educational attainment, particularly among those with only a primary school education (7).

Family support and functioning are among the key factors that can influence the mental health of women during pregnancy. A study conducted on 184 pregnant women showed that family dysfunction was significantly associated with increased levels of depression and anxiety, as well as decreased use of effective coping strategies such as planning and spiritual coping. However, demographic variables did not show a significant impact on mental health in this study (8).

Social support refers to the emotional (e.g., caring), informational (e.g., sharing important information), instrumental (e.g., assisting with housekeeping), tangible (e.g., providing financial aid), and/or psychological support provided by family members, friends, and community members (9). Social support from family, friends, and the community is linked to improved prenatal well-being (10). A systematic review and meta-analysis by Asres Bedaso et al. also confirmed that inadequate social support significantly increases the risk of antenatal depression and anxiety (11). A study by Kazemi et al. found that pregnant women who perceived greater social support were more likely to engage in health-promoting behaviors (12).

Despite numerous studies on the impact of social support on the mental health of pregnant women, the specific relationship between perceived social support and prenatal distress within particular cultural contexts, especially in Zanjan, remains underexplored. In recent years, significant social and cultural changes have occurred in Iran, which may affect family and community support structures and influence how pregnant women experience and cope with stress during pregnancy. The findings of this research can aid in developing culturally and socially tailored interventions to improve maternal mental health and advance knowledge in the field of perinatal mental health. The aim of this study is to examine the relationship between perceived social support and prenatal distress among pregnant women attending the prenatal clinic of Ayatollah Mousavi Hospital in Zanjan, located in northwestern Iran.

### Research hypothesis

Higher levels of perceived social support are significantly associated with lower levels of prenatal distress among pregnant women attending the prenatal clinic at Ayatollah Mousavi Hospital.

## Materials and methods

### Setting and design of study

This cross-sectional study was conducted on pregnant women who referred to the prenatal clinic of Ayatollah Mousavi Hospital in Zanjan city from March 2023 to the end of November 2023. Ayatollah Mousavi Hospital is a tertiary hospital has specialist antenatal clinics.

### Participants

#### Inclusion and exclusion criteria

The study included mothers aged 18 to 41 with singleton pregnancies in the gestational age over 20 weeks who were interested in participating. Exclusion criteria included chronic diseases (e.g., diabetes, hypertension), unintended or high-risk pregnancies, recent stressful events (like the death of close relatives within the last three months), use of psychotropic medications, and depression in the past six months. In Iran, mental health screening of pregnant women is routinely conducted to enable early identification of psychological disorders and timely intervention. This screening typically takes place during the first prenatal visit and includes assessment of psychiatric history, use of psychiatric medications, and evaluation of risk factors.

### Procedure

Eligible women were recruited through convenience sampling during their outpatient prenatal care visits at the Ayatollah Mousavi Hospital clinic. This approach was chosen due to practical constraints, including limited time and financial resources, which made randomized or stratified sampling impractical. In addition, the study’s specific inclusion and exclusion criteria required a targeted participant pool.

### Sample size

The sample size calculation for the correlation coefficient between social support and anxiety in pregnant mothers (r = 0.04), as reported by Fayazi et al., was conducted using an alpha level of 0.05 and a desired statistical power of 1 - β = 0.85. This analysis indicated that a minimum sample size of 220 participants was necessary to achieve adequate statistical power (13). The calculations were performed using G*Power software.

### Data instruments

#### Baseline data

Demographic questions included age, parity, gravida, education, occupation, and gestational age. Family monthly income was qualitatively assessed in three categories: insufficient for expenses, sufficient, and more than sufficient, based on self-report.

#### Prenatal Distress Questionnaire

The Pregnancy Distress Questionnaire, developed by Alderdice et al., comprises 12 questions across three subscales: concerns about childbirth and the baby, distress regarding weight and body image, and emotional and relational issues, specifically designed to measure prenatal distress. It employs five Likert scales with scores from 12 to 60, where a higher score indicates greater concern during pregnancy (14). The Persian version of this questionnaire had also been validated by the authors of Yousefi’s study and found to have very good internal consistency (Cronbach α = 0.80) (15). In the current study, the reliability (Cronbach’s alpha) of the Prenatal Distress Questionnaire was 0.84.

#### Social support questionnaire

Zimmet’s Perceived Social Support Questionnaire is a 12-item tool that assesses individuals’ perceptions of love and attention from family, friends, and others. Responses are rated on a seven-point scale from 1 (completely disagree) to 7 (completely agree), allowing for total scores ranging from 12 to 84, with subscale scores for family, social, and friends support ranging from 4 to 28. Higher scores indicate greater perceived social support. Preliminary psychometric evaluations revealed high internal consistency, with Cronbach’s alpha values of 0.91 for the overall scale, and 0.87, 0.83, and 0.98 for the family, social, and friends’ subscales, respectively. Test-retest reliability over a two-week period showed correlation coefficients of r=0.86 for the overall scale, r=0.78 for family support, r=0.69 for social support, and r=0.75 for friends’ support (16). In the current study, the reliability (Cronbach’s alpha) of Zimet’s Perceived Social Support Questionnaire was 0.87.

### Statistical methods

Data were analyzed using International Business Machines (IBM) SPSS Statistics version 26 (IBM Corp., Armonk, USA) at an alpha = 0.05 significance level. Mean and standard deviation, frequency, and percentage were assessed to display descriptive information for the demographic characteristics. The data were found to be non-normally distributed according to the Kolmogorov-Smirnov test. The associations among variables were explored using Spearman’s rank correlation coefficient. The Mann–Whitney U test and Kruskal–Wallis test were used to compare scores across different demographic characteristics. To identify predictors of prenatal distress and social support, a backward conditional logistic regression model was applied using median cut-off points for each variable, with a 95% confidence interval. The independent variables included in the backward conditional logistic regression model were age (years), parity (number of births), gravida (number of pregnancies), income level, education level, job status, and gestational age.

## Results

### Descriptive data

**Figure 1:**
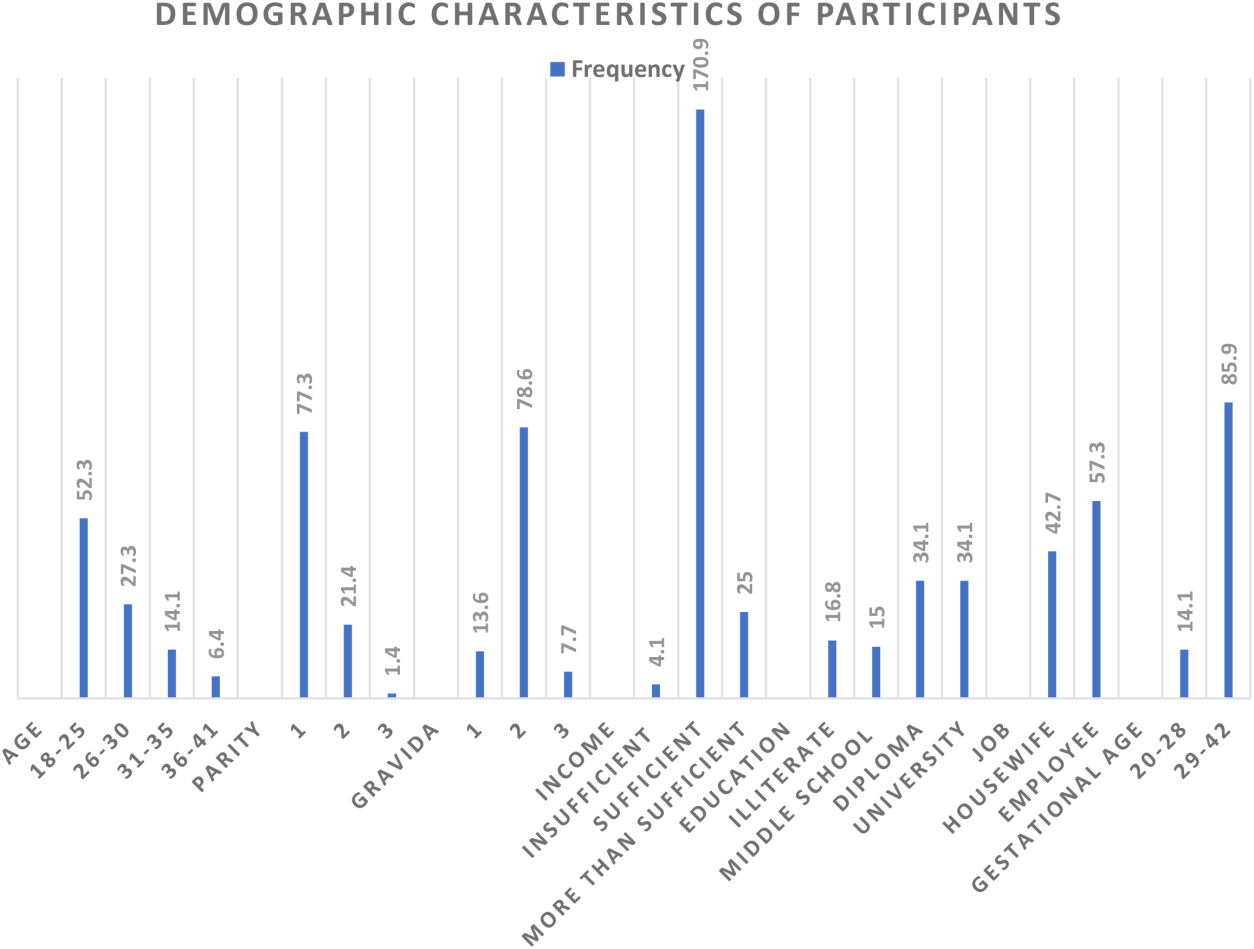
The demographic characteristics of participants.

In this study, 220 pregnant women were investigated. Most participants were pregnant women with a parity of 1, a gravida of 2, and a gestational age over 28 weeks. The majority were aged 18 to 25, held a diploma or higher education, and were employed. Most women had a monthly family income that was sufficient to cover their family expenses (**Figure 1**).

### Outcome data

Prenatal Distress and Social Support Description.

The mean overall perinatal distress score was 39.38 (SD = 6.04). The mean scores for specific concerns were as follows: concerns about childbirth and the baby, 17 (SD = 4.03); emotional and relational issues, 12.47 (SD = 3.83); and weight and body image, 9.9 (SD = 2.63).

The total social support score had a mean of 45.66 with a standard deviation of 10.91. The mean score for social support from others was 17.25 (SD = 6.17), from family was 12.47 (SD = 5.11), and from friends was 15.93 (SD = 5.12) (**Table 1**).

**Table 1:** Anxiety and social support scores in pregnant women participating in the study.

Variable		Mean	St. deviation	Minimum	Maximum
Prenatal Distress	Total Score	39.38	6.04	23	53
	Childbirth and The Baby	17.00	4.03	2	26
	Emotional Relational Issues	12.47	3.83	4	19
	Body Image	9.90	2.63	4	15
Social support	Total score	45.66	10.91	19	73
	Others	17.25	6.17	2	42
	Family	12.47	5.11	1	26
	Friends	15.93	5.12	1	26

### Main results

#### Comparison of demographic characteristics with perinatal distress

According to **Table 2**, the average of perinatal distress scores varied significantly based on parity and education level. Women with parity of one and those who were either illiterate or had a university-level education had higher distress scores (p<0.05).

**Table 2:** Comparison of prenatal distress scores based on demographic characteristics.

Variable		Mean (SD)	p-value	95% CI
Age(years)	18-25	39. 24)6.36)	0.194**	[38.067, 40.420]
	26-30	39.61(5.39)		[38.221, 41.011]
	31-35	38.12(6.09)		[35.892, 40.365]
	36-41	29.21(4.64)		[39.218, 45.353]
parity	1	39.90(6.02)	0.032**	[38.988, 40.812]
	2	37.87(5.86)		[36.151, 39.594]
	3	33.66(4.01)		[23.627, 43.706]
Gravida	1	38.33(5.16)	0.393**	[36.403, 40.263]
	2	39.42(6.34)		[38.469, 40.374]
	3	40.82(3.76)		[38.889, 42.757]
income	Insufficient for Expenses	36.11(5.71)	0.247**	[31.721, 40.500]
	sufficient	39.46(6.11)		[38.494, 40.429]
	More than Sufficient	39.69(6.04)		[38.114, 41.267]
Education	illiterate	41.81(5.38)	0.016**	[40.015, 43.606]
	middle school educated	38.09(5.33)		[36.199, 39.982]
	diploma	38.30(6.09)		[36.904,39.708]
	university-educated	39.82(6.28)		[38.380, 41.272]
Job	housewife	39.57(6.16)	0.684**	[38.312, 40.836]
	employee	39.23(5.97)		[38.184, 40.291]
Gestational age	20-28	32.19(5.41)	0.852*	[37.208, 41.179]
	29-42	39.41(6.15)		[38.529,40.295]

*Mann Whitney U test.

**Kruskal–Wallis test.

However, there were no significant differences in perinatal distress scores when comparing age, gravida, family income, occupation, or gestational age(p>0.05) (**Table 2**).

#### Comparison of demographic characteristics with social support

The average of social support scores varied significantly based on age, parity, gravida, income level, employment status, and gestational age. Women aged 18-25, with a parity of 3, a gravida of 3, a low-income level, who were housewives, or with a gestational age of 29–42 weeks, had higher social support scores. However, there were no significant differences in social support scores when comparing education levels (**Table 3**).

**Table 3:** Comparison of social support scores based on demographic characteristics.

Variable		Mean (SD)	p-value	95% CI
Age(years)	18-25	47.16(11.58)	0.033*	[45.026, 49.304]
	26-30	45.20(11.19)		[42.307, 48.092]
	31-35	44.16(6.24)		[41.870, 46.452]
	36-41	38.64(9.54)		[33.129, 44.155]
parity	1	44.59(10.59)	0.020*	[42.990, 46.197]
	2	49 (11.14)		[45.728, 52.271]
	3	54(15.52)		[15.435, 92.564]
Gravida	1	39(8.60)	0.001**	[35.786, 42.213]
	2	46.15(10.50)		[44.574, 47.726]
	3	52.47(13.23)		[45.666, 59.274]
income	low	56.66(13.44)	0.001**	[46.332, 67.001]
	Middle	44.06(11.11)		[42.306, 45.821]
	high	48.40(8.16)		[46.193, 50.606]
Education	illiterate	45.24(8.84)	0.898**	[42.292, 48.193]
	middle school educated	44.60(12.64)		[40.122, 49.089]
	diploma	46.26(12.21)		[43.456, 49.077]
	university-educated	45.73(9.74)		[43.490, 47.976]
Job	housewife	47.67(11.35)	0.018**	[45.343, 49.996]
	employee	44.16(10.35)		[42.340, 45.993]
Gestational age	20-28	41.41(12.52)	0.019*	[36.826, 46.012]
	29-42	46.35(10.49)		[44.853, 47.866]

*Mann Whitney U test.

** Kruskal–Wallis test.

#### Correlation between prenatal distress and social support

There were significant bivariate correlations between perceived social support and prenatal distress in pregnant women. Total social support was negatively correlated with total prenatal distress scores (r = -0.355, p= 0.001), as well as prenatal distress related to the childbirth and the baby (r = -0.472, p= 0.001) and emotional and relational issues (r = -0.119, p= 0.078), but not with prenatal distress related to body image (r = 0.090, p= 0.183).

Social support from others had a strong negative correlation with prenatal distress related to childbirth and the baby (r = -0.528, p = 0.001) and total prenatal distress (r = -0.447, p = 0.001). Social support from family had a moderate negative correlation with total prenatal distress (r = -0.359, p = 0.001) and prenatal distress related to emotional and relational issues (r = -0.255, p = 0.001). Social support from friends showed a significant negative correlation with prenatal distress related to body image (r = -0.149, p = 0.028) but was less strongly correlated with other prenatal distress dimensions (**Table 4**).

**Table 4:** Bivariate correlation between perceived social support and prenatal distress in pregnant women.

	Total social support	Others	Family	Friends	Total prenatal distress	Childbirth, the baby	Emotional, relational issues	Body image
Total Social Support	1							
Others	0.774*	1						
P-Value	0.001							
Family	0.548*	0.243*	1					
P-Value	0.001	0.001						
Friends	0.518*	0.121	0.017	1				
P-Value	0.001	0.073	0.806					
Total Prenatal Distress	-0.355*	-0.447*	-0.359*	-0.246*	1			
P-Value	0.001	0.001	0.001	0.001				
Childbirth and The Baby	-0.472*	-0.528*	-0.315*	0.087	0.811*	1		
P-Value	0.001	0.001	0.001	0.198	0.001			
Emotional Relational Issues	-0.119	-0.246*	-0.255*	0.399*	0.752*	0.476*	1	
P-Value	0.078	0.001	0.001	0.001	0.001	0.001		
Body Image	0.090	0.094	0.147*	-0.149*	-0.124	-0.352*	-0.498*	1
P-Value	0.183	0.165	0.030	0.028	0.066	0.001	0.001	

*Correlation is significant at the 0.05 level (2-tailed).

#### The predictive factors of prenatal distress and social support description

**Table 5** displays logistic regression analysis for Prenatal Distress and social support of pregnant women. All demographic variables were entered into the model and backward conditional logistic regression with median cut-off points were conducted. After adjusting for gravida, income, job, gestational age, age, and education, parity remained the only statistically significant variable in predicting Prenatal Distress, based on the seventh step of the model. Therefore, women with higher parity had a 0.462 times lower chance of experiencing prenatal distress.

**Table 5:** Logistic regression model analysis for Prenatal Distress and social support of pregnant women. .

Variables		B	OR	p-value	Nagelkerke R²	Wald test	95% CI
Prenatal Distress	Parity	-0.773	0.46	0.018	0.037	5.57	[0.243, 0.877]
Social support	Parity	0.88	2.42	0.007	0.131	7.36	[1.27, 4.51]
	Age	-0.48	0.61	0.003	0.44	0.84	[0.44, 0.84]

CI, confidence interval; OR, Odds Ratio.

In the analysis of social support, it was found that women with a higher parity experienced greater levels of social support (OR = 2.42, p=0.007). In contrast, increasing maternal age was significantly associated with lower levels of social support (OR = 0.61, p=0.003). To evaluate the explanatory power of the logistic regression models, we reported the Nagelkerke pseudo-R² values. The Nagelkerke R² values indicated that the model explained 3.7% of the variance in prenatal distress and 13% of the variance in social support.(**Table 5**).

## Discussion

This study aimed to assess the relationship between perceived social support and prenatal distress in pregnant women who were referred to the prenatal clinic of Ayatollah Mousavi Hospital in Zanjan City. Furthermore, the findings indicated significant negative correlations between the total perceived social support score and various dimensions of prenatal distress in pregnant women. Specifically, distress related to childbirth and the baby, as well as emotional and relational issues, showed significant inverse associations with social support. However, no significant correlation was found between social support and body image, related prenatal distress. Analysis of different sources of social support revealed that they moderated prenatal distress in varying ways. Social support from friends demonstrated a significant negative correlation with body image–related distress. Social support from others showed a strong negative correlation with distress associated with childbirth and the baby, whereas social support from family had a moderate negative correlation with distress related to emotional and relational issues. The results of the present study were consistent with the results of the study by Bahrami et al., 2022. They conducted a study in Babol and found that perceived stress and pregnancy distress negatively correlate with social support. Additionally, they showed that perceived stress, pregnancy distress, and low social support adversely affect pregnant women’s self-care. Importantly, social support was found to mediate these relationships (17). Olabisi et al. (2024) investigated how social support and body image perception affect psychological distress in pregnant women during their third trimester in Nigeria. Their findings indicate that negative body image perception is associated with increased psychological distress. They found that appraisal support reduces it by 1.9 points, belonging support decreases it by 2.1 points, and tangible support lowers it by 1.0 points (18).

Our study’s results differed from Olabisi ‘s study regarding the overall relationship between social support and body image in pregnant women. However, some specific areas of social support aligned with body image, indicating that each area of social support can have a distinct influence. This finding highlights the important role of social support in managing stress associated with weight and body image changes during pregnancy (18). Healthcare providers should design targeted interventions to enhance different types of social support (from friends, family, and others) based on the specific needs of pregnant women, particularly in areas such as emotional well-being, body image, and childbirth-related concerns.

Cultural differences, socioeconomic status, access to healthcare, and familial structures, can significantly impact the perception of body image, leading to varied levels of psychological distress. Jonsdottir et al. (2017) studied the impact of partner relationships and social support on women experiencing perinatal distress, characterized by symptoms of depression, anxiety, and stress. They found that women dissatisfied with their partner relationship were four times more likely to experience perinatal distress (1). The results of the present study are consistent with the results of the study of Jonsdottir et al.

Social support is crucial for reducing stress during life stages like pregnancy, when both psychological and physical changes occur (19, 20). In our study, social support from others accounted for the highest scores, whereas support from family was the lowest. Moreover, higher parity was significantly associated with increased odds of receiving social support, with women who had more previous births been 2.42 times more likely to perceive higher levels of social support compared to those with fewer or no previous births. Reversely according to the results of study of Fayazi et al. the most perceived social support was provided by the family and the least by the others (13). A study conducted by J. Peter et al. found a significant negative correlation between perceived social support and anxiety levels in pregnant adolescents (20). This means that higher levels of perceived social support are associated with lower levels of Prenatal Distress. These results were consistent with the results of our study.

Our results revealed that that younger women with higher education levels and more children were less likely to experience high levels of Prenatal Distress. However, there were no significant differences in perinatal distress scores when comparing age, gravida, family income, occupation, or gestational age. Women aged 18-25, with a parity of 3, a gravidity of 3, a low-income level, who were housewives, or with a gestational age of 29–42 weeks had higher social support scores. However, there were no significant differences in social support scores when comparing education levels. In contrast, Olabisi found a significant relationship between psychological distress and factors such as nuclear family type, self-employment, and secondary education level. Additionally, being married correlated with a reduction in psychological distress by an average of 0.2 points (18). Mohammadpour et al.’s study found a significant inverse correlation between perceived social support and perceived stress, while no significant relationships were observed between socio-demographics and perceived stress (21). The differences in findings between our study and above studies may be due to variations in cultural, socioeconomic, and demographic factors. This finding highlights the importance of context on prenatal distress and social support. Prenatal care programs should incorporate psychological assessments and social support evaluations as part of routine care to identify at-risk women and provide early interventions to reduce prenatal distress.

### Strengths of the study

One strength of our study is the use of a dedicated questionnaire for assessing perinatal distress, combined with a robust sample size.

### Limitations

This study has several limitations. First, its cross-sectional design prevents establishing causal relationships between variables. Future studies using longitudinal designs are recommended to enable the assessment of temporal relationships and strengthen causal inferences. Second, the use of convenience sampling may introduce selection bias and limit the representativeness and generalizability of the findings. Therefore, results should be interpreted with caution. Future studies should employ more rigorous sampling methods, such as random or stratified sampling, to improve representativeness and external validity. Third, the data were collected from a single center, which may restrict the applicability of the results to other settings or populations. Conducting multi-center studies across diverse geographic and demographic contexts would improve the external validity of the findings. Finally, although participants with known psychiatric disorders or those receiving psychiatric treatment were excluded, the study did not comprehensively control for all potential confounding variables, such as undiagnosed pre-existing mental health conditions, which may have influenced the outcomes. Future research should incorporate more detailed psychiatric assessments to better control for these confounders. The logistic regression models explained a relatively small proportion of the variance in prenatal distress and social support, indicating that other important factors influencing these outcomes were not captured. Future research should include a broader range of variables to improve the explanatory power of predictive models.

## Conclusion

This study found moderate levels of prenatal distress and perceived social support among pregnant women. The most common distress domains were concerns about delivery and the infant’s health, while perceived support, particularly from family was inversely correlated with overall distress. Parity emerged as the only independent predictor of both distress and social support. These findings suggest that first-time mothers may be more vulnerable to prenatal distress and could benefit from enhanced social support interventions. Further research is needed to better understand the mechanisms by which parity influences both distress and social support to inform more effective prenatal care programs.

## Data Availability

The original contributions presented in the study are included in the article/supplementary material. Further inquiries can be directed to the corresponding author.
